# Self-Assembled Polymers for Gastrointestinal Tract Targeted Delivery through the Oral Route: An Update

**DOI:** 10.3390/polym15173538

**Published:** 2023-08-25

**Authors:** Xiaoyu Yang, Yang Yang, Haiyan Yu, Yi Zhou

**Affiliations:** 1 Department of Pharmacy, Union Hospital, Tongji Medical College, Huazhong University of Science and Technology, Wuhan 430030, China; 2 Pingshan General Hospital, Southern Medical University, Shenzhen 518118, China; 3 Pingshan District Peoples’ Hospital of Shenzhen, Shenzhen 518118, China

**Keywords:** self-assembled, polymers, oral delivery, biomedical applications, GIT targeting

## Abstract

Gastrointestinal tract (GIT) targeted drug delivery systems have gained growing attention as potential carriers for the treatment of different diseases, especially local colonic diseases. They have lower side effects as well as enhanced oral delivery efficiency because of various therapeutics that are vulnerable to acidic and enzymatic degradation in the upper GIT are protected. The novel and unique design of self-assembled nanostructures, such as micelles, hydrogels, and liposomes, which can both respond to external stimuli and be further modified, making them ideal for specific, targeted medical needs and localized drug delivery treatments through the oral route. Therefore, the aim of this review was to summarize and critically discuss the pharmaceutical significance and therapeutic feasibility of a wide range of natural and synthetic biomaterials for efficient drug targeting to GIT using the self-assembly method. Among various types of biomaterials, natural and synthetic polymer-based nanostructures have shown promising targeting potential due to their innate pH responsiveness, sustained and controlled release characteristics, and microbial degradation in the GIT that releases the encapsulated drug moieties.

## 1. Introduction

In recent decades, there has been a global increase in the prevalence of intestinal diseases, including irritable bowel syndrome, colorectal cancer (CRC) [1], and inflammatory bowel disease (IBD), which comprises Crohn’s disease and ulcerative colitis [2]. CRC is responsible for the highest number of cancer-related deaths in Europe, with over 200,000 deaths annually, while the incidence of IBD is rising at an alarming rate in previously low-incidence areas, such as Asia [2]. Moreover, ulcerative colitis has been recognized as one of the modern refractory diseases by the World Health Organization (WHO) [3]. Therefore, effective treatments of intestinal diseases have become major public health issues worldwide.

Oral administration is the most commonly used method for drug delivery due to its simplicity, high safety, and lower cost compared to intravenous injection. It also promotes strong patient compliance. Additionally, drugs administered orally can quickly reach the GIT, enabling accumulation in the target site of the intestine, which makes it an ideal delivery route for intestinal-targeted therapies [4]. However, effective treatments for intestinal diseases still present several challenges, such as poor oral absorption, difficult gut location, and inadequate drug delivery [5]. Expectations for accumulation in the targeted sites, multiple physiological barriers (including the enzymatic barrier, mucus layer barrier, and intestinal epithelial cells layer) in the GIT also hinder the effectiveness of drugs in the gut [6,7]. The enzymatic barrier, rooted in an abundance of digestive enzymes in the GIT, can cause the degradation of some drugs [8]. The mucus layer can hinder the penetration of charged or hydrophobic drugs, while the lipophilicity of intestinal epithelial cell membranes can impede the absorption of hydrophilic drugs through transcellular pathways [9]. In addition, the oral bioavailability of insoluble drugs is often extremely low because the drug molecules need to be dissolved or highly dispersed in the stomach and intestinal fluid to be absorbed [10]. To tackle these challenges, an ideal drug delivery system should be capable of preserving the activity of drug molecules by protecting them against degradation, improving their solubility, mitigating their toxicity and other biological side effects, penetrating in vivo barriers such as the epithelium and cell membrane, specifically targeting cells, and controlling drug release.

The colonic site of the GIT has been identified as an optimal site for oral drug delivery due to its near-neutral pH and longer transit time, which result in lower proteolytic enzyme activity and greater responsiveness to absorption enhancers. These factors make the colon a favorable site for delivering various drug molecules, including proteins and peptides [11]. To achieve effective colon-specific drug delivery, it is necessary to prevent drug release in the upper part of the GIT and to have a triggering mechanism that allows for abrupt drug release upon reaching the colon [12]. Nowadays, researchers have developed several strategic approaches based on overcoming multiple barriers in the GIT for gut-targeted drug delivery, especially colon targeting [13]. Various primary approaches for colon-specific delivery, such as pro-drugs [14], pH-sensitive polymers [15], timed-release delivery systems [16], and microbially degraded delivery systems, have been achieved through the self-assembly of different materials. Among these strategies, drug delivery systems have received significant attention. 

Drug delivery systems, such as nanoparticles (NPs), provide a versatile and scalable platform for encapsulating a wide range of drugs and ensuring efficient therapeutic outcomes, especially in the treatment of gastrointestinal diseases [17]. NPs exhibit enhanced stability, ensuring the preservation of drug integrity during transit through the GIT, including acidic pH and enzymatic degradation [18]. Additionally, NPs enable controlled and targeted drug release, minimizing off-target effects and enhancing therapeutic efficacy. The convenient construction of NPs allows for the incorporation of diverse drugs, regardless of their physicochemical properties, expanding therapeutic options [19]. In comparison to other types of NPs, self-assembled drug delivery systems exhibit a remarkable level of structural and functional complexity, which greatly enhances their ability to overcome gastrointestinal barriers efficiently. This enables effective absorption of drugs and their distribution to target tissues, thereby improving both bioavailability and therapeutic outcomes [20]. Furthermore, the simplicity of self-assembled synthesis allows for various decoration methods to achieve gastrointestinal targeting [21]. Although self-assembled nanoparticles have obvious advantages compared with other nanoparticles, there are still only a handful of products that have been approved for market, and a large number of oral self-assembled nanoparticles are still in the clinical trial or preclinical research stage. MMX mesalamine (LIALDA, USA) is the first FDA-approved once-daily oral formulation of mesalamine, which is based on MMX Multi Matrix System^®^ (MMX^®^) technology by Cosmo Pharmaceuticals. MMX technology allows the controlled delivery of mesalamine throughout the entire colon. In Phase III studies, it was found that MMX mesalamine achieved a complete mucosal healing rate of 32% after 8 weeks of treatment compared to 16% in the placebo group, with similar frequencies of adverse events observed in both groups [22]. These results indicate that MMX Mesalamine is an advantageous treatment for moderate ulcerative colitis. Amecolo, utilizing the MMX^®^ technology, is the pioneering FDA-approved antibiotic designed specifically for the treatment of traveler’s diarrhea [23]. By directly targeting rifamycin to the colon, Amecolo effectively circumvents undesired systemic side effects. To sum up, further research and development in this field holds great promise for advancing therapeutic strategies and improving patient outcomes.

In this review, self-assembled nanostructures, or what we mainly called NPs, for intestinal delivery are summarized, with an emphasis on the intelligent response performance of the carriers. The challenges of oral targeted delivery are expounded upon, and the advantages of intestinal-targeted NPs are described. Subsequently, the design concept and synthesis methods are reviewed, and various polymer materials that can be used to construct self-assembled NPs are systematically summarized to shed light on further developments for applications in intestinal therapies.

## 2. Self-Assembled Drug Delivery System

Self-assembled drug delivery systems are effective in shielding the drug from digestive enzymes in the GIT, thereby preventing its degradation and enhancing its stability [24]. Furthermore, the nano- or micro-scale particles facilitate the uptake of drugs by intestinal epithelial cells [25], which helps to overcome both the enzymatic and physical barriers that impede the oral absorption of drugs [26]. Moreover, carriers can provide high drug loading capacity. Importantly, surface modification of the nano-drug delivery system can further enhance its uptake efficiency or targeting efficiency, thereby effectively improving the oral bioavailability or bio-distribution in the target sites of drugs [27,28]. Due to these advantages, self-assembled drug delivery systems present excellent application prospects in intestinal diseases therapy. 

A self-assembled drug delivery system is typically composed of natural or synthetic polymer materials that are capable of encapsulating or adsorbing drugs to produce loaded drugs’ NPs [29]. The self-assembly of NPs provides a simple and reproducible way to achieve unique and enhanced properties for various applications [30]. Supramolecular biomaterials have many benefits, such as modularity, mechanical tenability, biomimicry, responsiveness, and dynamic reciprocity. Self-assembled materials can offer high levels of precision molecular control, providing a unique opportunity to generate well-controlled structures from a diverse array of molecular building blocks [31]. These materials can create new tools to probe biochemical interactions and design guidelines for new therapy strategies, such as triggering responses through selective molecular pathways.

Molecular self-assembly is a process where noncovalent, weak interactions between molecules drive their assembly and organization, resulting in supramolecular structures that define the material’s properties [31]. The self-assembly method has been mainly utilized to construct micelles and vesicles from lipids and polymers for delivery purposes. However, other structures, such as tubules, fibrils, or complex systems like molecular hydrogels can be prepared via self-assembly. Monomeric building blocks can be designed to self-assemble spontaneously or assemble in response to an exogenous stimulus, yielding materials whose bulk properties are defined at the molecular level by the monomer. Consequently, the release profiles and mechanical properties of these materials can be tailored specifically for their intended use by appropriate monomer design. Additionally, in some cases, the therapeutic agent can be present when self-assembly is triggered, allowing for the direct encapsulation of precise concentrations of the drug within the supramolecular structure, offering a distinct advantage concerning the loading of the therapeutic.

## 3. Preparation of Self-Assembled Nanostructures

Self-assembling molecules are often amphiphilic, containing both hydrophobic and hydrophilic domains. The hydrophilic part can be charged (anionic, cationic, or zwitterionic) or uncharged. The separation of hydrophobic and hydrophilic domains in space leads to the molecule’s amphiphilicity. Amphiphilic molecules, such as lipids, peptides, and proteins, form the basis of biological structures, such as cell membranes, cytoskeletons, and extracellular matrices. Lipids are one of the simplest amphiphilic structures. Under aqueous conditions, these amphiphilic molecules associate through weak non-covalent interactions to form ordered assemblies ranging in size from nanometers to micrometers. The thermodynamic driving force for most self-assembly events is provided by the desolvation, collapse, and intermolecular association of the monomer hydrophobic parts. Polarity-based intermolecular interactions, such as electrostatic and hydrogen bonding, also contribute to the dynamics and specificity of the structure. The final assembled structure depends on the structure of the monomer and the external environment in which self-assembly occurs. Self-assembly is a dynamic process that can be triggered or reversed by external stimuli, providing the possibility to form assemblies before delivery and to decompose the final supramolecular structure after delivery to the target site. Amphiphilic molecules self-assemble into various nano- and micro-sized structures in an aqueous solution, including micelles, vesicles, and molecular gels composed of tubes, fibrils, and fibers. Micelles are composed of a hydrophobic core surrounded by a shell of solvated hydrophilic groups. Amphiphiles with intermediate hydrophobicity preferentially assemble into bilayer vesicles, which are spherical, hollow, sheet-like structures surrounding an aqueous core. Layered structures also include water-filled tubes and sheets. Hydrogels are one of the largest structures that can be achieved by self-assembly, consisting of three-dimensional continuous networks surrounded by a liquid aqueous phase. Purely self-assembled hydrogels are non-covalently crosslinked, and their mechanical integrity depends on the network structure’s nodes and entanglements. This paper aims to provide an overview of self-assembling molecules in the application of GIT targeting (especially colon targeting) through the oral route, highlighting the potential of these materials for encapsulating and delivering therapeutics. Specifically, examples of recent progress in the literature will be given according to the different polymers used.

## 4. Polymers from Natural Materials

Polymers applied in the self-assembled NPs can be classified into two major types based on their origin: natural polymers and synthetic polymers [32]. Both types have been used in the preparation of orally delivered self-assembled NPs. Natural polymers are generally ideal for fabrication because of their better biocompatibility with the natural environment, ability to respond to certain environmental stimuli, degradability, and low toxicity potential. Several natural polymers have been used for NPs through the oral route targeting the GIT, such as polysaccharides (such as chitosan, alginate, and hyaluronic acid,) and proteins (such as gelatin and heparin). As these natural polymers have many advantages over synthetic ones, they are preferred for drug delivery applications. However, they lack adequate mechanical properties and tensile strength, making it difficult to tune them to drug delivery needs. Moreover, they are natural to the body’s environment, so they also have the potential to trigger immunological responses that compromise their use in biomaterials for oral drug delivery. Synthetic polymers, such as polypeptides, polyesters, and polyphosphazene, usually offer controllable degradation rates and better mechanical strengths compared to natural polymers. However, in most cases, both natural and synthetic polymers cannot offer desirable properties when used alone, so a combination of both is used for the effective designing of NPs for colon (GIT) targeting and treatment, with improved properties.

### 4.1. Polysaccharide

Polysaccharides are biological macromolecules formed by monosaccharide units linked through glycosidic bonds. They offer advantages such as high biocompatibility, ease of functional modification, and wide availability. Consequently, polysaccharide provides unique application advantages and potentials in the development of oral delivery systems for gut targeting. As shown in Table 1, chitosan and alginate are two of the polymers which have been studied most for the oral delivery.

#### 4.1.1. Chitosan and Its Derivatives

Chitosan is a kind of natural alkaline amino polysaccharide that removes part of the acetyl group from chitin. Compared with other kinds of polysaccharides, the chemical structure of chitosan is easily modified. Modified chitosan is widely used in self-assembled nanodrug delivery systems. Yan et al. [33] designed TMC-NPs and SA-TMC-NPs (SA represents alginate decoration) as drug delivery systems for low molecular weight heparin (LMWH) using trimethyl chitosan (TMC). The results revealed that these two NPs could significantly increase the accumulation of LMWH in the inflamed colon and exert remarkable anti-colitis effects on the colonic mice due to their excellent colon targeting property. Alginate decoration lets NPs have stronger mucoadhesion with lower oral absorption, which resulted in better effects of SA-TMC-NPs on colitis than TMC-NPs. Janardhanam et al. [34] prepared a layer-by-layer film with chitosan and sodium alginate polyelectrolytes containing either 5-fluorouracil (5FU) or moxifloxacin HCl (MF). Comparing with intravenous injection, oral administration of these two NPs led to a higher accumulation of loaded drugs (5FU for 2.37 times and MF for 7.10 times) in the colon tissues. Self-assembly polymeric NPs made of naphthyl-grafted succinyl chitosan (NSC), octyl-grafted succinyl chitosan (OSC), and benzyl-grafted succinyl chitosan (BSC) were used to deliver 19-tert-butyldiphenylsilyl-8,17-epoxy andrographolide (3A.1, a kind of anticancer drug) [36]. These 3A.1-loaded NPs possessed high drug loading capacity (160.27 ± 10.03 μg/mg) and negatively charged surfaces. These NPs were stable in the simulated gastric fluid (pH 1.2), while drugs could release in the simulated intestinal fluid (pH 6.8), which suggested that NPs had the potential for oral anticancer drug delivery to colorectal cancer cells. 

Otherwise, decorated chitosan is found to have an excellent adsorption ability in the intestinal tract, which leads to the accumulation of drugs in the gut. Liu et al. designed a kind of nanoparticle that which possessed a core of insulin and TMC and a “mucus-inert” surface decorated with N-(2-hydroxypropyl) methacrylamide copolymer derivative (pHPMA). The NP showed remarkable ability for mucus penetration and epithelium permeation, which meant that the NP was promising for enhancing the number of drugs that reached target cells in the gut. Zhang’s work [39] developed a self-assembled polyelectrolyte complex NP by coating insulin-loaded dodecylamine-graft-γ-polyglutamic acid micelles with TMC. The NP could enhance its affinity with intestinal epithelial cells because of the modification of its goblet cell-targeting peptide, which revealed its potential to target the gut. 

Looking ahead in the field of chitosan and its derivatives for oral delivery, several potential developments are worth considering. The application of nanotechnology holds great promise for improving the performance of chitosan carriers, including solubility, stability, and bioavailability [52]. Additionally, harnessing the interaction between chitosan and gut microbiota for regulating drug delivery offers new avenues for personalized therapies [53]. Moreover, the development of multifunctional chitosan carriers aims to achieve precise drug delivery through strategies such as targeted ligands, controlled release systems, and co-delivery of drugs, while achieving personalized treatments through customized delivery systems tailored to individual patient needs will drive further innovation in oral drug delivery [54]. With continued scientific advancements, these trends hold promise for breakthroughs and advancements in the field of oral delivery.

In summary, chitosan and its derivatives possess positively charged surfaces and abundant amino and hydroxyl groups in their chains, which are beneficial for mucus penetration and epithelium permeation. Thus, modified chitosan is a prospective material for targeting enteric diseases. 

#### 4.1.2. Sodium Alginate

Sodium alginate is a natural polysaccharide derived from brown seaweed with an anionic linear structure composed of (1–4) linked β-D-mannuronic acid and α-L-guluronic acid residues. It has the advantages of biocompatibility, biodegradability, and non-toxicity, making it suitable for drug delivery. 

Kohli et al. [55] fabricated a nanoparticulate system (BNPs) using alginate and chitosan polymers to improve the oral bioavailability of berberine (BBR), a phytogenous alkaloid. The BNPs had a size of approximately 202.2 nm, a zeta potential of −14.8 mV, and an encapsulation efficiency of 85.69%, which indicates excellent stability and a high drug-loading capacity. As ex vivo gut permeation studies showed, BNPs exhibited effective penetration through cells and intestine. Importantly, compared to BBR suspension, BNPs provided a remarkable 4.1-fold increase in the oral bioavailability of BBR on a female Wistar rats’ model. Li et al. [56] developed alginate microbeads incorporating chitosan nanoparticles (CNP) to achieve controlled release of insulin. The release of insulin from the microbeads was found to be pH-dependent, with acidic pH retarding protein release and neutral pH promoting it. When administered to hyperglycemic mice, the CNP demonstrated a significant reduction in blood glucose levels compared to mice treated with insulin-free alginate microbeads. Notably, the blood glucose-lowering effects persisted for up to 96 h, highlighting the controlled release capability of CNP for insulin. Ayub et al. [37] used layer-by-layer (LbL) self-assembly to formulate a self-assembled, cysteamine-based, disulfide cross-linked sodium alginate that was designed to deliver paclitaxel (PCX) to colonic cancer cells. The PCX-loaded nanospheres exhibited an excellent encapsulation efficiency (77.1%) and a sustaining drug release (45.1%). More than 70.0% of the nanospheres were successfully internalized into HT-29 cells, indicating the nanospheres can effectively permeate through the mucus. This work revealed that these nanospheres might be considered as potential carriers for targeted drug delivery to the colon. However, permeation ability through the intestinal epithelial layer barrier of nanospheres and drug release behaviors in vivo has not been investigated yet, so the consistency of behavior in vivo and in vitro remains questionable. 

#### 4.1.3. Cyclodextrin 

Cyclodextrin is a generic term for a series of cyclic oligosaccharides produced by amylose under the action of cyclodextrin glucosyltransferase by Bacillus, including α-cyclodextrin, β-cyclodextrin, γ-cyclodextrin, and so on. Cyclodextrin polymers can be formed by chemical bonding, physical mixing, and group inclusion with cyclodextrin molecules. Cyclodextrin polymers not only have the special inclusion complex function of cyclodextrin cavity, but also possess the strong stability and adjustability of physical and chemical properties.

NPs [40] based on β-cyclodextrin (named AON) containing a pro-resolving annexin A1-mimetic peptide (Ac2-26) were developed to treat inflammatory bowel disease (IBD). The results revealed that AON could effectively reduce symptoms of inflammation and reshape the gut microbiota composition. These effects are attributed to the increment of Ac2-26′s release and accumulation at the specific inflammatory sites in response to high levels of ROS on the IBD mouse model. Meanwhile, AON remained stable in the GIT and showed excellent safety profiles on the mouse model. Bai [41] et al., developed RG@M-γ-CD CNPs forming by host–guest complexation and self-assembling of mannose-modified γ-cyclodextrin (M-γ-CD) with Regorafenib (RG). The pharmacokinetics and bio-distribution experiments reflected that RG@M-γ-CD CNPs could effectively accumulate at the colorectal neoplasms on the colon cancer mouse model. It was proven that RG@M-γ-CD CNPs could target tumor-associated macrophages (TAMs) and specifically inhibited TAM activation, which led to a strong anti-inflammatory effect and anti-tumor effect. Catenacci et al. [42] conjugated inulin with β-cyclodextrin (β-CD) and formed a self-assembly drug carrier (INUCD). Inulin was colon degradable, which led to a directed release of loaded drugs by INUCD. Meanwhile, inulin itself is beneficial to the well-being of the colon. Results revealed that INUCD, with a narrow size distribution of 381 nm and a zeta potential of −9.4 mV, could perfectly achieve the colonic targeted delivery of hydrophobic drugs without permeating the epithelial layer. Xue et al. [44] synthesized a polyethylene glycol (PEG) block and a hydrophilic copolymer containing α-cyclodextrin (α-CD) or β-CD blocks. As model guest compounds, poly(β-benzyl L-aspartate) and pyrene were used to form Tpl/PEG-P(bCD) NPs by host–guest interaction-mediated self-assembly. Results showed that the drug loading content of NPs could be up to 18.0 ± 3.0% and have an affinity-controlled drug release. NPs exhibited desirable anti-inflammatory and antioxidant effects on macrophages with high bio-safety. Furthermore, NPs could effectively accumulate in the inflamed colon in the mice colitis model, which indicates promising applications in targeted therapy for enteric diseases.

#### 4.1.4. Glycosides 

Glycosides are a class of natural organic compounds composed of a sugar moiety and a non-sugar moiety that are widely distributed in nature. The sugar moiety of glycosides can be a monosaccharide, disaccharide, or polysaccharide, while the non-sugar moiety can be a fatty acid, phenol, alkaloid, and others. As natural products, glycosides exhibit good biocompatibility and biosafety, which can effectively reduce toxic side effects. Moreover, chemical modifications on sugar or non-sugar moieties of glycosides can adjust their site-specificity, biological activity, solubility, stability, and degradation, which can optimize drug performance and enhance therapeutic efficacy. 

Shen et al. [45] prepared doxorubicin (DOX) and superparamagnetic iron oxide (SPION)-loaded solid lipid NPs (SLNs) coated with folate (FA) and dextran. DOX/SPIONs-loaded DFSLNs were proven to accumulate at the colon tumor sites. The SLNs were decorated with FA residues and coated with a dextran shell layer, which served to evade cellular transport and systemic absorption by avoiding biorecognition with the proton-coupled FA transporters located on brush border membrane surfaces in the small intestine. Additionally, the dextran coating enhanced accumulation in the colon through a specific association with dextranase, which was present exclusively in the colon. After enzymatic degradation and removal of the dextran shells, the SLNs targeted tumors on a cellular level through complementary binding with FAR-overexpressed CT26 colon cancer cells. This resulted in facilitated cellular uptake of the DFSLNs by the cancer cells via FAR-mediated endocytosis, leading to pronounced antitumor effects from the chemo/magnetothermal combination therapy of the SLN formulations, both in vitro and in vivo. Notably, the DFSLNs demonstrated effective inhibition of orthotopic colon tumor growth in tumor-bearing mice receiving SLNs’ treatments, referring to the prominent therapeutic efficacy of DFSLNs for local treatment by oral administration without apparent systemic side effects. Bertoni et al. [46] prepared an oxidation-responsive dextran-modified polymer (OxiDEX) and formed chitosan-decorated rifaximin-NPs by the nanoprecipitation method. The drug release of OxiDEX NPs was highly relevant to physiological hydrogen peroxide in the GIT, while the chitosan coating ensured their stability and provided intestinal adhesion. These advantages suggested that OxiDEX NPs can be a potential targeted therapy of IBD through the pH and ROS responsiveness. Similarly, Lee [47] et al. designed pH and ROS-responsive NPs by blending dextran-drug conjugate (Nap−Dex) with the acid-sensitive acetylated dextran polymer (Ac-Dex). It was discovered that drugs could be fully released within 20 min in a simulated inflammatory environment (10 mM H_2_O_2_). In addition, naproxen-loaded NPs were found to reduce IL-6 levels by 120 times and TNF-α levels by 6 times more efficiently than free naproxen in activated macrophages. These results showed a possibility of NPs for treating intestinal inflammation. Deka et al. [48] prepared nanoassemblies through self-assembled Azo-AG 5 conjugates. The neomycin aminoglycoside portion and 4-dimethylamino azobenzene respectively form hydrophilic shells and a hydrophobic core, while eosin and aspirin are loaded in the core. Nanoassemblies with positive surface charge disintegrated regularly at pH 4 and showed a mobility on agarose gel, which indicated that they might possess excellent colon adhesion.

#### 4.1.5. Others 

Johnson et al. [51] constructed self-assembled microparticles by grafting ferulic acid onto fructo oligosaccharide, resulting in the formation of self-assembled disc-shaped microparticles of approximately 2 μm in size. Both HA-adh-DOCA and HA-adh-GA showed high drug loading capacities (DL) of silybin (up to 20%) and displayed similar steady continued-release patterns in simulated gastrointestinal fluids and PBS. Single-pass intestinal perfusion studies indicated that silybin-loaded micelles were absorbed in the whole intestine and transported via a passive diffusion mechanism. More importantly, the conjugation of ferulic acid with oligosaccharide could alleviate antioxidant stress on HT29 and LoVocell models (colon cancer cell lines) due to their excellent antioxidant ability. These studies revealed that HA-adh-DOCA was a good candidate for alleviating colorectal cancer. However, the effects of HA-adh-DOCA on the colon cancer animals could be further investigated.

### 4.2. Peptide and Protein

Peptides or proteins have shown potential for the preparation of NPs with enhanced efficacy, such as high loading capacity, safety, and functional properties [57]. Comparatively speaking, peptides and proteins derived from plants are less expensive and more hydrophobic than animal sources, which are more conducive to NPs’ formation. In particular, low molecular weight is beneficial for maintaining integrity in harsh conditions in the GIT. In recent years, researchers have focused on applications of natural peptides and proteins as drug delivery systems and explored their targeting effects in the GIT, as shown in Table 2. 

#### 4.2.1. Proteins

Proteins exhibit good biocompatibility and high biological recognition, thereby improving the targeting and therapeutic efficacy of nanocarriers. However, protein carriers face several problems, such as low drug loading capacity, difficulties in preparation and modification, and susceptibility to environmental factors such as pH value and temperature. Pointed chemical decoration can effectively expand the application of protein carriers.

Zein-based self-assembled NPs are reported to have potential for oral delivery due to their natural hydrophobicity, edibility, biodegradability, and biocompatibility [57]. Pauluk et al. [65] developed chitosan-coated zein NPs which showed excellent mucoadhesive properties in vitro. The resveratrol loaded NPs had a mean particle size of 173.4 nm and a zeta potential of −22.7 mV, indicating their great stability in the simulated gastric fluid (SGF) and simulated intestinal fluid (SIF). However, one limitation of the study is that the in vitro evaluation of mucoadhesive properties may not accurately reflect the in vivo behavior of the NPs, and the pharmacokinetics and pharmacodynamics of the NPs can be further investigated. Wang’s [66] work proposed a new preparation method for zein NPs by heat-induced self-assembly, which was simple, fast, and low-cost. A series of self-assembled zein NPs were prepared in aqueous ethanol through thermal treatment at 70 °C, and their physicochemical properties were evaluated. These NPs exerted good stability at neutral or acidic conditions and could decrease drug release rates in vitro, which indicated their potential application as oral drug delivery systems. This study only focused on NPs’ properties in vitro; given the lack of data on animal models, subsequent studies are needed to further evaluate the biocompatibility and drug delivery effects.

Jiang et al. [63] conjugated linoleic acid (LA) with β-lactoglobulin (β-LG) to form cis-9 loaded complex self-assembly (β-LG-CLA). The complex exerted great stability at pH conditions in the GIT and enhanced transport through the Caco-2 monomer (nearly 3 times) which was attributed to the lipocalin character of β-LG. Moreover, the cis-9 loaded complex exhibited better treatment than CLA on the colon cancer cell model. All these experiments indicated that β-LG had the potential to be a colon-targeted carrier. Regrettably, the effects on the animal models were not described in this work. The release dynamics of CLA from β-LG-CLA complex in gastrointestinal conditions and specific binding sites could be explored further in order to precisely control release.

Du et al. [62] prepared self-assembled NPs based on natural silk fibroin (SF). The patchouli alcohol (PA)-loaded SF NPs decorated with cyclo RGD peptide (cRGD–PASFNs) showed great stability, controlled release, release, and bio-safety. As cRGD could be recognized specifically by integrin αv, cRGD–PASFNs exhibited notably targeted drug delivery to the inflamed colon because of up-regulated integrin αv expression in the colitis tissues. The CT imaging identified that cRGD–PASFNs effectively accumulated in the colon. The cRGD–PASFNs accelerated the relief of inflamed colon in colitis mice, which showed a lower disease activity index than the control group after treatments for 10 days. Biochemical tests revealed that cRGD–PASFNs restored the functions of barriers in the GIT by modulating amino acid levels relevant to intestinal mucosal repair and regulating immune response, thereby accelerating relief of the inflamed colon in colitis mice. This work evaluated cRGD–PASFNs at animal and cellular levels. 

#### 4.2.2. Peptides

Peptides have a smaller molecular weight than proteins, resulting in smaller particle sizes and higher surface area, which allows for easier penetration into tissue gaps and intracellular compartments, improving drug permeability and bioavailability. However, peptides have limitations, such as lower drug loading capacity and susceptibility to enzymatic degradation, oxidation, and heat denaturation, which necessitate modification or combination with other materials to improve stability, which limits their applications for drug delivery.

Shi et al. [59] utilized ε-poly-L-arginine (EPL) to functionally modify insulin (INS), resulting in self-assembling into a core-shell structure nanosphere (INS@EPL). Meanwhile, both targeting peptide and PEG were decorated on INS@EPL. PEG decoration protected enzymolysis, while targeting peptides located the intestine. The remained insulin percent increased nearly 4.5 times due to the encapsulation of INS@EPL with low coating density (INS@EPL-L) after incubation with trypsin for 4 h, and the protective effects improved with the increase in EPL decoration density (INS@EPL-H). This concentration-dependent phenomenon also occurred in the cell penetration and animal tests. The apparent permeability (*P_app_*) values of INS@EPL-L and INS@EPL-H were significantly increased compared with free insulin (5.0 times and 12.0 times, respectively). Administration of INS@EPL-H at 20 IU/kg exerted more effective hypoglycemic effects in diabetic rats for up to 12 h. This study exhibited a promising carrier for the oral delivery of insulin. The metabolism and bio-distribution of insulin nanospheres can be further investigated to better understand their behavior and safety in vivo. 

### 4.3. Lipid Cyclodextrin

Lipid cyclodextrin is a type of cyclodextrin molecule modified with lipids, possessing the structural characteristics of cyclodextrin and the specific properties of lipids. Compared to cyclodextrin, lipid cyclodextrin undergoes lipid modification on its structure, enabling it to exhibit both the inclusion capability of cyclodextrin and the solubility and lipophilicity of lipids.

From Table 3, we can see that lipid nanoparticle formulations are commonly used for oral delivery of poorly water-soluble drugs. Studies have shown that lipid excipients may stimulate bile secretion and the release of digestive enzymes, and the resulting lipid digestion products may play a crucial role in aiding the dissolution of drug molecules and promoting their absorption [67].

#### 4.3.1. Acylglycerols

Acylglycerols are a class of compounds composed of fatty acids and glycerol, which are one of the main components of fats. They are commonly known as triglycerides (TG) or triacylglycerols (TAG) and are naturally occurring lipids in animals and plants. The good biocompatibility of acylglycerols makes them a favorable candidate as a drug delivery carrier. However, its storage stability in vitro is limited, which hinders its application.

Sung et al. [70] identified three major lipids, including phosphatidic acid, monogalactosyldiacylglycerol, and digalactosyldiacylglycerol, in ginger. They were mixed in an orderly fashion at the ratio of 5:2:3 in phosphate-buffered saline (PBS) to form new lipid NPs (nLNPs) by self-assembly. The IL-22 mRNA-loaded nLNPs (200 nm, −18 mV) could up-regulate the protein expression level of IL-22 in the colonic mucosa of colitis mice. Furthermore, nLNPs promoted the recovery of colon length, colonic MPO activity, and correlated inflammatory markers, which led to an accelerated recovery from acute colitis. This study suggested that nLNPs could be excellent mRNA delivery platforms for targeting ulcerative colitis. The clear mechanisms of nLNPs can be further studied in the follow-up work.

#### 4.3.2. Steroid Lipids

Steroid lipids are a class of lipids that contain a steroidal nucleus, including cholesterol, steroid hormones, and steroidal amines. Like other lipids, steroid lipids are easily oxidized in vivo. The susceptibility of steroid lipids to oxidation in vivo represents a drawback that hinders their use as drug carriers. It is feasible to use approaches such as co-administration with antioxidants or physicochemical modifications to alter their susceptibility to oxidation in order to mitigate the oxidation challenge.

Dioleoylphosphatydic acid (DOPA) and cholesterol at the ratio of 4:1 were vortexed with the probiotic bacterium *Escherichia coli Nissle 1917* (EcN) in calcium phosphate buffer to formulate lipid-membrane-coated bacteria by biointerfacial, supramolecular self-assembly [71]. This platform, named LCB, presented significantly improved survival of bacterium against the gastrointestinal environment without changing their viability and bioactivity. In the STm-induced mouse model of colitis, the ratio of EcN/STm in feces excreted from mice treated with LCB was 25 to 8000 times higher than uncoated EcN, which suggested that LCB could effectively protect EcN and promote the function of EcN to improve the intestinal immunity. Similar results were confirmed on DSS-induced colitis. This study proposed a unique platform by self-assembled lipid membranes for bacteria oral delivery. However, the morphology and number of bacteria encapsulated in the lipid membranes produced cannot be precisely controlled, which may affect their therapeutic efficacy and safety. Additionally, the biosafety of this new platform can be meaningful for further study in the future.

#### 4.3.3. Phospholipids

Phospholipids are a group of lipid molecules that make up the main component of cell membranes, consisting of a hydrophilic phosphate glycerol head group and two hydrophobic fatty acid tails. Phospholipids can self-assemble into various nanocarriers. These nanocarriers have found widespread applications in drug delivery, gene therapy, and molecular imaging due to their excellent biocompatibility, stability, and drug loading capacity. As a result, they hold great promise as effective vehicles for targeted therapy and efficient drug delivery.

Shanmugam et al. [72] developed a nanocochleates (CPT) system encapsulating paclitaxel (PTX) by phosphatidylserine to treat resistant colon cancer by oral administration. The CPT degraded less at an acidic pH, while it continued to be released at intestinal pH for 48 h, which meant it led to a stable intestinal release of PTX. CPT-PTX exhibited excellent anticancer effects on HCT-116 & HCT-15 cells (multi-drug resistant). Surprisingly, after oral administration of CPT-PTX for 5 days’, tumor growth inhibition was up to 25 times higher compared with intravenous Taxol on the HCT-15 drug-resistant colon cancer xenograft mouse model. This study provided a novel nano-spirochaete that could effectively improve the bioavailability and efficacy of PTX. The distribution and metabolism in vivo have not been fully studied, so further exploration is needed.

### 4.4. Nucleic Acid and Its Derivates

Nucleic acid is a naturally occurring biological molecule that exhibits excellent biocompatibility and biosafety, making it unlikely to cause immune or toxic reactions. Due to its inherent negative charge, nucleic acids can interact with drugs through electrostatic and other chemical reactions, resulting in high drug loading and stable encapsulation on NPs, as shown in Table 4.

Lin et al. [74] summarized the research status of nucleic acid as nanocarrier materials and approved potential applications in controlled and targeted therapies. This summary enlightened us whether nucleic acid could be used for oral GIT targeted delivery. Nie et al. [73] fabricated nanocrystal self-assembled microspheres by nicotinamide riboside (NR) and resveratrol (RES) nanocrystal. The NR/RESms showed a sustained release in the SIF when it possessed a limited release in the SGF, which indicated the potential of the intestinal target. 

### 4.5. Other Natural Products

In addition to the normal natural polymers such as polysaccharide, peptide, lipid and nucleic acid, other products like phospholy-deoxycholate di-adenosine, glycyrrhizic acid, and epicatechin have also been applied in oral delivery, as shown in Table 5.

Yiguang Jin and colleagues [10] synthesized a new prodrug called zidovudinephosphoryl-deoxycholyl didanosine (ZPDD), which combines zidovudine (AZT) and didanosine (ddI) in one molecule. This bolaamphiphilic prodrug showed amphiphilicity according to monolayers at the air–water interface and self-assembled into spherical vesicles in water. Intravenous administration of ZPDD to mice demonstrated good targeting ability as it was found in macrophage-rich tissues in vivo and rapidly released AZT in the targeted tissues. The bioavailability of ZPDD was 30.8% for oral administration compared with the venous route, indicating that it may be a promising nanomedicine for delivering two types of drugs to targeted tissues orally. Another type of micelles was developed by Chengying Shen and colleagues [11] using the glycyrrhizic acid (GL) to deliver the water-soluble monoterpene glucoside- Paeoniflorin (Pae) through the oral route. Pae-loaded GL micelles were prepared using the ultrasonic dispersion method. After optimization, it was shown to exhibit delayed drug release and a significantly higher permeability of Pae in the duodenum (*p* < 0.05), jejunum (*p* < 0.05), ileum (*p* < 0.01), and colon (*p* < 0.01) intestine after perfusion of Pae-loaded GL micelles compared to Pae solution. Furthermore, the Cmax and AUC0–t values of Pae-loaded GL micelles were nearly 2.18- and 3.64-fold more developed after encapsulation. These results indicate that GL-loaded micelles have the potential to be used for oral delivery of Pae.

## 5. Polymers from Synthetic Materials

Self-assembly of NPs through natural molecules provides a simple and reproducible method to enhance properties, such as permeability from chitosan and long-term adhesion for alginate, that play a crucial role in drug delivery through the GIT [78,79,80]. Synthetic molecules, on the other hand, offer several benefits, such as modularity, mechanical tenability, biomimicry, responsiveness, and dynamic reciprocity, making them a good candidate for self-assembled NPs [25,52,53,54]. Compared to natural molecules, self-assembled polymers from synthetic molecules offer high levels of precise molecular control, providing a unique opportunity to generate well-controlled structures from a diverse array of molecular building blocks.

### 5.1. PEGs

PEG is a water-soluble non-ionic polymeric molecule that exhibits superior biocompatibility, lower toxicity, and lower immunogenicity compared to other polymers. As a result, it is widely used in various fields, particularly in the biomedical field, for drug delivery [81,82,83], as shown in Table 6.

HS15 has been shown to improve the oral bioavailability of several lipophilic drugs. Jing-Wen Luo et al. [84] developed a self-assembled nanomicelle for the oral administration of nimodipine (NIM), which has poor water solubility. This nanomicelle improved the cellular transport of NIM in Caco-2 cell monolayers, ultimately enhancing the intestinal absorption of NIM by 3.13- and 2.25-fold in the duodenum and jejunum, respectively, one hour after oral administration to rats. Clathrin, lipid raft/caveolae, and macropinocytosis mediated the cell uptake of NIM nanomicelles, while P-glycoprotein and endoplasmic reticulum/Golgi complex (ER/Golgi) pathways were involved in exocytosis. Similarly, Haiyue Long et al. [85] prepared another self-assembled nanomicelle using amphipathic mPEG-PLLA to deliver pyrinezolid through the oral route. In vivo, studies demonstrated that PZ-M had a prolonged blood circulation time and increased oral bioavailability compared to free PZ. Pyrinezolid-loaded mPEG-PLLA nanomicelles were prepared through a one-step solid dispersion process, and PZ exhibited sustained release as a result of encapsulation within the inner PLA cores of the micelles. 

One of the most important applications of self-assembled PEG-based NPs should be in the treatment of inflammatory bowel disease (IBD) via the oral route. Shanshan Li et al. [14] fabricated a self-assembled and oxidation-degradable Janus-prodrug, Bud-ATK-Tem (B-ATK-T), consisting of ROS-responsive aromatized thioketal (ATK) linked to the anti-inflammatory drug budesonide (Bud) and antioxidant tempol (Tem). Relying on the hydrophobic interactions and π-π stacking interactions of ATK, prodrug B-ATK-T successfully self-assembled into NPs with lecithin and DSPE-PEG2K. Unlike the simple co-administration of the free drugs, self-assembled B-ATK-T NP with extensive ROS sensitivity could release both Bud and Tem simultaneously and proportionately. In vitro, studies have shown that B-ATK-T NP could exert excellent responsive-release characteristics and the combined efficacy of anti-inflammation, anti-oxidation, and anti-apoptosis in inflammatory cells. Strikingly, B-ATK-T NP can accumulate in the DSS-induced inflamed mice colon and effectively increase the maximum drug concentration while also avoid potential systemic side effects. B-ATK-T NP inhibited the expression of oxidative and proinflammatory mediators more effectively than free drugs, and also significantly reduced death caused by IBD.

Xinyu Wang et al. [87] have also contributed to the treatment of IBD through oral routes using PEG-based NPs. They developed a self-assembled supramolecular nanoparticle based on natural polyphenol tannic acid and poly(ethylene glycol) containing polymer for oral antibody delivery. The nanoparticle can be aqueous assembled without organic solvent and scaled up to gram level easily due to its pH-dependent reversible assembly ability. Oral administration of antibody-loaded nanoparticles achieved high accumulation in the inflamed colon and low systemic exposure. The novel formulation of anti-TNF-α antibodies administrated orally achieved high efficacy in the treatment of colitis mice compared with free antibodies that were administered orally. The polyphenol-based supramolecular nanoparticle is a promising platform for the oral delivery of antibodies for the treatment of inflammatory bowel diseases with promising clinical translation prospects.

### 5.2. Surfactant

A surfactant-assisted cooperative self-assembly is a promising approach to achieving well-defined active nanomaterial structures and properties by confining the NP self-assembly or growth within nano-compartments formed by amphiphilic surfactants in aqueous solutions [93]. Amphiphilic surfactants are molecules that have hydrophilic groups (heads) and hydrophobic groups (tails) and which can form aggregates, such as micelles, in aqueous solutions. Surfactant-assisted cooperative self-assembly can facilitate the growth of hydrophobic nanoparticles or precursors within the hydrophobic interiors of micelles, leading to the formation of highly ordered superstructures. This process involves the growth from nanoparticles to seed structures and then to final complex 1D–3D nanoparticle active nanostructures, resulting in uniform and complex structures. Recent advances in this field have led to the synthesis of new classes of highly ordered active nanostructures with diverse properties and applications. As shown in Table 7, researchers have achieved significant progress in the surfactant-assisted cooperative self-assembly of NPs, leading to the development of new classes of highly ordered active nanostructures.

Heba M. Aboud et al. [94] have made significant progress in developing a thermodynamically and kinetically stable mixed polymeric micelle system for oral delivery of the sparingly soluble drug valsartan (VAL). The micelles were prepared using 2/1.75% *w*/*v* of PF127/TW80 through the thin film hydration method. The relative bioavailability of VAL-loaded mixed micelles was found to be 3.75 times higher than VAL suspension after oral administration. In rat models, the nanomicellar form of VAL was found to be more effective at protecting against cardiac injury induced by cisplatin when either pre- or simultaneously administered with cisplatin. The molecular mechanism for this protection was found to be two signaling pathways, Mhrt/Nrf2 and Trx1. These findings demonstrate the potential of mixed polymeric micelles for improving the oral delivery of sparingly soluble drugs like VAL, and their potential for use in the treatment of various diseases.

Emad B. Basalious et al. [59] have developed nanocolloidal polymeric micelles for the delivery of lapatinib, a highly hydrophobic drug used to treat breast cancer. These micelles have a uniform, spherical nanosize of below 150 nm, which makes them useful for passive tumor targeting using the EPR effect. The combination of Soluplus^®^ with PF127 has provided the system with thermodynamic and kinetic stability, and also explained the resistivity of the LP-PMs to dilution in a large pool of body fluids. Therefore, this system can be successfully employed as a drug carrier for both oral and intravenous administration.

### 5.3. Others

Several other polymers, such as Eudragits [12], are commonly used as pH-dependent coating polymers to achieve enteric or colon targeting of drugs. These systems are designed based on the assumption that the pH of the human GIT increases gradually from the stomach (pH 2.0–3.0), to the small intestine (pH 6.5–7.0), to the colon (pH 7.0–8.0), although recent studies have shown that the pH drops slightly in the colon and is highest in the ileocecal junction. Therefore, the polymer used for colon targeting should be able to withstand the lower pH value of the upper GIT and disintegrate at the neutral or slightly alkaline pH of the terminal ileum, preferably the ileocecal junction. Eudragit offers an effective solution to avoid drug release in the upper part of the GIT because it has a range of dissolution pH profiles across its polymer grades. However, the conventional colonic delivery system based solely on Eudragits may not be reliable in vivo due to the inherent variability of pH and emptying times from the GIT. To achieve controlled release of the drug from the coated system, a combination of pH-dependent polymers with polymers exhibiting time-based release has been proposed, as shown in Table 8.

Arnau Biosca et al. [100] developed PBMA-MESBMA-based NPs through direct dispersion in water of amphiphilic polymers, leading to the self-assembly of micellar structures. These NPs have been shown to penetrate the intestinal epithelium, making them suitable for oral administration. In vivo assays demonstrated significantly increased curcumin concentration in the blood of mice one hour after oral administration of PBMA-MESBMA-curcumin compared to the administration of free drug (18.7 vs. 2.1 ng/mL, respectively). The key factor in the formation of these NPs was the addition of a voluminous group in the quaternary amine of the zwitterionic block. This voluminous group increased solvation, reduced interchain/intrachain interactions, and decreased the hydrophilic character of the copolymer, favoring the formation of the simplest self-assembled nanoparticle. The introduction of a voluminous group also affected the area of the hydrophilic block, producing changes in the nanoparticle structure due to steric impedance.

Types of self-assembled NPs that combine not only efficient epithelial absorption but also mucus permeation have been reported by Wei Shan et al. [101], which could be applied for efficient oral delivery of insulin. These NPs contain a nanocomplex core consisting of insulin and cell penetrating peptide (CPP) and a hydrophilic coating of N-(2-hydroxypropyl) methacrylamide copolymer (pHPMA) derivatives that can dissociate from the nanoparticle surface as it permeates through mucus, revealing the CPP-rich core for subsequent transepithelial transport. The NPs exhibit 20-fold higher absorption than free insulin on mucus-secreting epithelium cells, and, when orally administered, generate a significant hypoglycemic response and an increase in serum insulin concentration in diabetic rats. These findings suggest that these NPs have the potential for efficient oral delivery of insulin.

## 6. Conclusions and Perspectives

Nowadays, several kinds of new techniques and materials have been found and applied for the oral delivery of drugs in the treatment of GIT-related diseases, such as colon cancer, ulcerative colitis, and IBD, as well as the enhancement of drug bioavailability through GIT. One of the most promising methods for such a purpose is self-assembly method using different polymers, which can produce a simple way to obtain the desired nanostructures, mainly NPs, efficiently.

In this review, the different materials for preparing self-assembled nanostructures applied for oral GIT targeting have been presented and discussed, including both natural products and synthetic molecules. Natural molecules provide a simple and reproducible method but might have a lower possibility of modification. Synthetic molecules provide more precise molecular control, which means better controlled-release effect as well as more distinct application. A major hurdle in GIT targeted nanoparticles studies is the lack of precise animal models (especially for GIT-related diseases, such as IBD, UC, or colon cancer). Although there are various animal models available, most studies focus on chemically induced models. While these models are convenient for studying drug delivery platforms, researchers should explore a wider range of animal models to fully translate biomaterial-based drug delivery to clinical practice [3,12,105]. In addition, patients with GIT diseases experience numerous physiological changes compared to healthy individuals, including pH variations in the GI tract, alterations in gut microbiota, and fluctuations in colonic enzymes. Standardizing these methods would enable better comparisons between research groups and improve the correlation between in vitro and in vivo situations in humans. Additionally, the models used to assess mucoadhesion and mucopenetration of nanodrug delivery are typically performed on mucus from healthy animals [106]. From a technological perspective, there are numerous obstacles associated with the complex manufacturing processes of nanoparticles, scalability challenges, and limited reproducibility [107]. These challenges can be addressed by leveraging the significant advancements in the field of nanomedicine over the past two years. While several limitations exist in this review, the main mechanism in the preparation of self-assembled nanostructures by different polymers has not been discussed clearly. Further comprehensive reviews to cover the different mechanisms involved in different materials as well as their effects on the drugs for GIT targeting could be another field to be discussed, which could provide more suggestions for oral delivery by the self-assembly method.

## Figures and Tables

**Table 1:** Polysaccharide in self-assembled nanostructures.

**Chitosan and Sodium Alginate**				
**Materials**	**Products**	**Applications**	**Years**	**Reference**
Chitosan	NPs	Ulcerative colitis	2020	[33]
Chitosan and sodium alginate	Assembled film	Colon targeting	2022	[34]
Paclitaxel, Trimethylchitosa, Folate	Drug conjugates	Cancer therapy	2017	[35]
Naphthalyl grafted succinyl chitosan, octyl grafted succinyl chitosan and benzyl grafted succinyl chitosan	pH-sensitive amphiphilic chitosan NPs	Colorectal cancer	2018	[36]
Sodium alginate	Nanospheres	Colon cancer	2019	[37]
Trimethyl chitosan, N-(2-hydroxypropyl) methacrylamide copolymer derivative	NPs	Enhanced mucus permeation	2016	[38]
Trimethyl chitosan, dodecylamine-graft-γ-polyglutamic acid	NPs	Enhanced bioavailability and prolonged hypoglycemic response	2015	[39]
**Cyclodextrin**				
**Materials**	**Products**	**Applications**	**Years**	**Reference**
β-cyclodextrin	NPs	Therapy for IBD	2019	[40]
γ-cyclodextrin	NPs	CRC treatment	2021	[41]
β-cyclodextrin	Bioconjugate	Local drug release	2020	[42]
β-cyclodextrin	Supramolecular self-assemblies	Targeted tumor therapy	2020	[43]
β-cyclodextrin/α-cyclodextrin	Affinity NPs	Ulcerative colitis	2018	[44]
**Glycosides**				
**Materials**	**Products**	**Applications**	**Years**	**Reference**
Dextran	Solid lipid nanoparticle	Colon tumors	2019	[45]
Dextran	Nano-in-micro composites	IBD	2018	[46]
Dextran	Dual-responsive NPs	Anti-inflammatory	2018	[47]
Neomycin, 4-dimethylaminoazobenzene	Cationic amphiphilic stimuli-responsive azobenzene-aminoglycoside conjugate	Colon-specific delivery and gene delivery	2015	[48]
**Others**				
**Materials**	**Products**	**Applications**	**Years**	**Reference**
Hyaluronic acid deoxycholic acid and hyaluronic acid glycyrrhetinic acid	Hyaluronic acid-glycyrrhetinic acid conjugate	Liver diseases therapeutics	2016	[49]
6-O-(3-cetoxy-2-hydroxypropyl)-hyaluronic acid	NPs	Cancer	2013	[50]
Fructooligosaccharide	Microparticle	Colorectal cancer	2020	[51]

**Table 2:** Peptide and protein in self-assembled nanostructures.

Materials	Products	Applications	Years	Reference
Waterborne bone morphogenetic protein 2	Artificial proto-osteocells	Bone tissue repair and regeneration	2018	[58]
Polylysine	Nanospheres	Oral therapeutic protein delivery	2015	[59]
Tetrapeptide	Nanostructures	Tumor	2017	[60]
Lactoferrin Conjugated linoleic acid	Micelles	Oral therapeutic protein delivery	2020	[61]
Natural silk fibroin	Silk fibroin NPs	Ulcerative colitis	2022	[62]
β-lactoglobulin (β-LG)	Bovine β-LG conjugated linoleic acid complex	Colon cancer	2010	[63]
Sodium polyaspartate	Nanogels	Gut inflammation	2019	[64]

**Table 3:** Lipid cyclodextrin in self-assembled nanostructures.

Materials	Products	Applications	Years	Reference
Liposome	NPs	Oral delivery	2011	[68]
Phospholipids, bile salts and cholesterol	Double-loaded bilosomes	Oral delivery	2022	[69]
Phosphatidic acid, monogalactosyldiacylglycerol, and digalactosyldiacylglycerol	NPs	Ulcerative colitis	2022	[70]
Dioxy phosphonic acid and cholesterol	Supramolecules	Oral delivery	2019	[71]
Phosphatidylserine	Nanocochleates system	Colon cancer	2020	[72]
Lecithin	NPs	IBD	2019	[14]

**Table 4:** Nucleic acid and its derivates in self-assembled nanostructures.

Materials	Products	Applications	Years	Reference
Nicotinamide ribose, Resveratrol	Microspheres	Improved oral NAD+ bioavailability	2021	[73]
Phospholipids, bile salts and cholesterol	Double-loaded bilosomes	Oral delivery	2022	[74]

**Table 5:** Other natural products in self-assembled nanostructures.

Materials	Products	Applications	Years	Reference
Zidovudine phospholy-deoxycholate diadenosine	Spherical vesicles	AIDS	2011	[75]
Glycyrrhizic acid	Micelles	Oral delivery	2021	[76]
Therapeutic samarium (Sm3+) ions and (-)-epicatechin (EC)	NPs	Colon cancer	2019	[77]

**Table 6:** PEG in self-assembled nanostructures.

Materials	Products	Applications	Years	Reference
HS15 PEG lithium dodecyl stearate	Nanomicelle	Oral delivery	2016	[84]
PEG	Micelles	Pneumonia	2017	[85]
Lecithin and PEG	NPs	IBD	2019	[14]
Polycaprolactone-polyethylene glycol	Micelles	Oral Squamous Cell Carcinoma	2021	[86]
PEG	NPs	IBD	2020	[87]
Azo-PEG-OMe	Conjugate	Oral delivery	2017	[83]
Gd-PEG-H1	Supramolecular nanostructured adjuvants	Tumor immunotherapy.	2019	[88]
Polyethyleneimine-poly (D, L-lactide) and 1,2-distearoyl-sn-glycero-3-Phosphoethanolamine-N-[methoxy (polyethylene glycol)	NPs	Colorectal cancer	2021	[89]
Copolymers of monomethoxy poly(ethylene glycol)-poly(ε-caprolactone) (MPEG-PCL)	Nanomicelles	Colon cancer	2015	[15]
PEGylated pterostilbene	Micelles	IBD	2023	[90]
Methoxy polyethylene glycol-polycaprolactone polymer	Micelles	siRNA oral administration	2022	[91]
PEG PLGA	Micelles	Ulcerative colitis	2022	[92]

**Table 7:** Surfactant in self-assembled nanostructures.

Materials	Products	Applications	Years	Reference
Pluronic F127 and Tween 80	Micelles	Cardiovascular diseases	2020	[94]
Pluronic/Phosphatidylcholine/polysorbate 80	Micelles	Cardiovascular diseases	2015	[95]
Soluplus^®^, Pluronic^®^F127	Micelles	Tumors	2020	[96]
Poloxamer P188	NPs	Breast cancer	2015	[97]
Pluronic F127 and Pluronic L121	Hydrogels	Tumors	2022	[98]

**Table 8:** Other synthesized products in self-assembled nanostructures.

Materials	Products	Applications	Years	Reference
Eudragit (R) S100	NPs	Colon cancer	2019	[99]
Butyl methacrylate—Morpholine ethyl sulfobetaine methacrylate (PBMA-MESBMA)	NPs	Plasmodium falciparum-infection	2021	[100]
N-(2-hydroxypropyl) methylacrylamide copolymer (pHPMA) derivative	NPs	Diabetes	2015	[101]
Polyether	Micelles	Ulcerative colitis	2022	[102]
polylactic acidpolyethyleneimine and hyaluronic acid-inuli	NPs	Colon cancer	2022	[103]
Ethylene glycol dimethacrylate	Hydrogel	Targeted drug release at colonic site	2019	[104]

## Data Availability

No new data were created or analyzed in this study. Data sharing is not applicable to this article.
